# Cost-effectiveness of Rotavirus vaccination in Vietnam

**DOI:** 10.1186/1471-2458-9-29

**Published:** 2009-01-21

**Authors:** Sun-Young Kim, Sue J Goldie, Joshua A Salomon

**Affiliations:** 1 Program in Health Decision Science, Department of Health Policy and Management, Harvard School of Public Health, Boston, MA, USA; 2 Department of Global Health and Population, Harvard School of Public Health, Boston MA, USA; 3 Harvard Initiative for Global Health, Cambridge, MA, USA

## Abstract

**Background:**

Rotavirus is the most common cause of severe diarrhea leading to hospitalization or disease-specific death among young children. New rotavirus vaccines have recently been approved. Some previous studies have provided broad qualitative insights into the health and economic consequences of introducing the vaccines into low-income countries, representing several features of rotavirus infection, such as varying degrees of severity and age-dependency of clinical manifestation, in their model-based analyses. We extend this work to reflect additional features of rotavirus (e.g., the possibility of reinfection and varying degrees of partial immunity conferred by natural infection), and assess the influence of the features on the cost-effectiveness of rotavirus vaccination.

**Methods:**

We developed a Markov model that reflects key features of rotavirus infection, using the most recent data available. We applied the model to the 2004 Vietnamese birth cohort and re-evaluated the cost-effectiveness (2004 US dollars per disability-adjusted life year [DALY]) of rotavirus vaccination (Rotarix^®^) compared to no vaccination, from both societal and health care system perspectives. We conducted univariate sensitivity analyses and also performed a probabilistic sensitivity analysis, based on Monte Carlo simulations drawing parameter values from the distributions assigned to key uncertain parameters.

**Results:**

Rotavirus vaccination would not completely protect young children against rotavirus infection due to the partial nature of vaccine immunity, but would effectively reduce severe cases of rotavirus gastroenteritis (outpatient visits, hospitalizations, or deaths) by about 67% over the first 5 years of life. Under base-case assumptions (94% coverage and $5 per dose), the incremental cost per DALY averted from vaccination compared to no vaccination would be $540 from the societal perspective and $550 from the health care system perspective.

**Conclusion:**

Introducing rotavirus vaccines would be a cost-effective public health intervention in Vietnam. However, given the uncertainty about vaccine efficacy and potential changes in rotavirus epidemiology in local settings, further clinical research and re-evaluation of rotavirus vaccination programs may be necessary as new information emerges.

## Background

Rotavirus is the most common cause of severe diarrhea leading to hospitalization or disease-specific death among children under 5 years of age [1,2]. The rotavirus infection is reported to cause more than 2 million hospitalizations and about 527,000 deaths annually (as of 2004), and the burden of disease is higher in developing countries [1]. Human rotavirus infections are characterized by the following features: (1) diverse genotypes that vary geographically and over time [3-5]; (2) frequently asymptomatic presentation or non-specific clinical symptoms (e.g., varying degrees of diarrhea, vomiting, or fever); (3) age-dependency of clinical manifestation (e.g., rotavirus infections in infants younger than 3 months old are often not severe) [6]; (4) common reinfections and varying degrees of protection against subsequent infections depending on the number of previous infections [7,8]; and (5) seasonality of incidence (e.g., rotavirus infections peak during the winter season typically) [9].

Since rotavirus is endemic in both developed and developing countries, improved hygiene is unlikely to be effective in reducing rates of infection, prompting great interest in the development of an effective vaccine [10,11]. Recently, two new oral rotavirus vaccines, Rotarix^® ^and RotaTeq^®^, have been approved [11,12]. To inform decision-makers in countries considering the introduction of rotavirus vaccine into their national immunization programs, a number of studies have attempted to quantify the health and economic impact of these vaccines in different settings, using model-based approaches [13-24]. Three studies have evaluated the impact of introducing rotavirus vaccines in low-income countries in Asia (one each in Vietnam [13], Uzbekistan [15], and Asia as a whole [14]) where the burden of rotavirus disease is greatest.

Estimating the avertable rotavirus disease burden is challenging since, despite some known characteristics described above, many aspects of rotavirus infection remain unknown. For example, although natural infections are reported to confer some level of immunity against subsequent infections, little is known about the specific nature of such immunity (e.g., relative strength and length of immunity compared with that from vaccines). There is also high uncertainty around the incidence of infection, as rotavirus infection is often asymptomatic, and even symptomatic cases can only be diagnosed definitively through laboratory testing, which is not usually performed even in medical facilities in developed countries [16]. For these reasons, most previous studies have estimated the avertable disease burden through vaccination based only on the estimated incidence of symptomatic rotavirus diarrhea (not rotavirus infection itself) and proportions of severe cases requiring medical treatment or leading to deaths, based on surveillance data [13-19,23,24]. Further simplifying assumptions are typically: one episode of rotavirus diarrhea at maximum, and full protection against subsequent rotavirus diarrhea during the first 5 years of life of a birth cohort [13-15,17,19,23]. The potential impact of the dimensions that are not incorporated in previous models on the cost-effectiveness of rotavirus vaccines have received less attention thus far.

Such uncertainty may be higher in the studies performed in low-income country settings, given that surveillance systems are relatively less comprehensive than in developed countries and that there are limited data from developing countries on local vaccine efficacy and safety. For example, although surveillance systems in some resource-poor countries provide some information on the incidence of rotavirus gastroenteritis requiring hospitalization, attributing outpatient visits to rotavirus infection is difficult since surveillance programs usually do not include outpatient clinic settings in those countries. While there is a concern that vaccine efficacy may be different from setting to setting partly due to the diversity and variability of rotavirus genotypes, in Asia and Africa Phase III clinical trials have only been recently initiated, and local vaccine efficacy and safety data will likely not become available until 2010 [25]. Indeed, the three studies performed in Asian settings [13-15] have used the efficacy data from clinical trials performed in other regions such as Europe or Latin America [26] without adjustment for the different genotype distribution.

In this paper we aimed to extend previous studies by developing a more detailed natural history model of rotavirus infection that uses the best available data, reflects the key features of rotavirus epidemiology in developing countries, incorporates reinfection and natural immunity, and comprehensively explores uncertainty surrounding these features. We applied the model to Vietnam in order to reevaluate the cost-effectiveness of a rotavirus vaccination program in this country.

## Methods

### Analytic overview

Since our focus was on developing countries, we chose to evaluate the Rotarix^® ^vaccine, which is an attenuated human rotavirus vaccine targeting the highly prevalent G1P[8] genotype, introduced in 2004 and tested through phase III clinical trials in some Latin American countries [26,27]. For our analysis, following the clinical definition of severe gastroenteritis (an episode of diarrhea requiring overnight hospitalization or rehydration therapy in a medical facility) used in a phase III clinical trial of Rotarix^® ^[26], we assumed that the rotavirus infection leads to one of the following infection outcomes: asymptomatic, mild gastroenteritis (requiring home care only), or *severe *gastroenteritis (requiring an *outpatient visit *or *hospitalization *or leading to *disease-specific death*). Based on our working definitions, we constructed a Markov model (TreeAge Pro 2006) that simulates the acquisition of infection, immunity, and resolution of symptoms. We applied the model to the 2004 Vietnamese birth cohort (N = 1,644,000) [28] and followed the cohort through age 5 to reevaluate the cost-effectiveness of introducing the rotavirus vaccine into Vietnam, from both the societal (base-case) and healthcare system perspectives. We chose Vietnam as our example in part because of the availability of epidemiological and cost data, and in part to compare our results to a previous study performed in Vietnam [13]. Model outcomes included cases of outpatient clinic visits and hospitalization, rotavirus diarrhea-associated deaths, disability-adjusted life years (DALYs), and costs incurred during the first 5 years of life of the cohort. Primary results were expressed as the incremental cost (2004 US dollars) per DALY averted of rotavirus vaccination compared to no intervention. To facilitate the interpretation of cost-effectiveness results, we consider interventions with incremental cost per DALY averted less than a country's GDP per capita 'very cost-effective' and interventions with the corresponding ratios between 1 and 3-times the GDP per capita 'cost-effective', as suggested by the World Health Organization (WHO) [29]. For the base-case, both effectiveness and cost were discounted at 3%. To explore parameter uncertainty, we performed a multivariate probabilistic sensitivity analysis as well as univariate sensitivity analyses.

### Model

**Figure 1:**
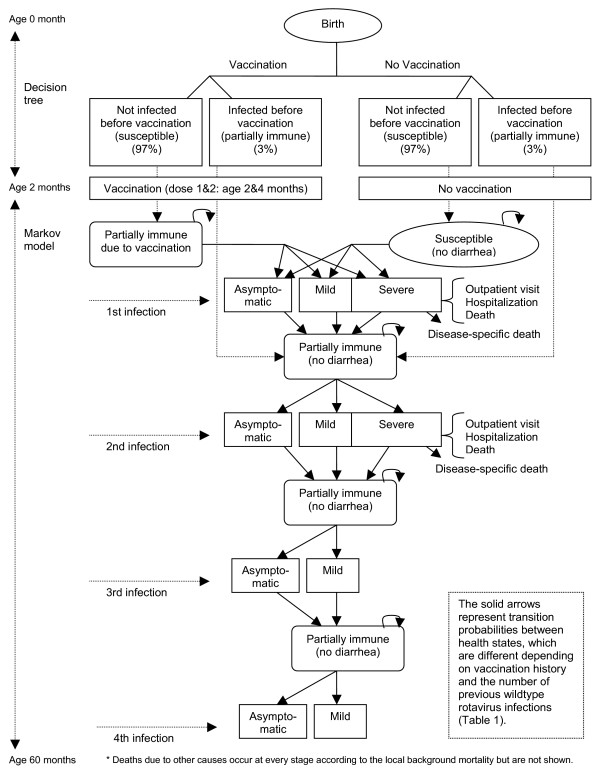
**Model schematic**. This figure presents the schematic of the natural history of rotavirus infection. Natural infection by wild type rotavirus can provide protection against subsequent infections with a varying degree of immunity depending on the number of previous infections as well as ages at infection.

In order to allow rotavirus reinfection, distinguish primary rotavirus infection from subsequent infections with different levels of partial immunity, and account for the timing of vaccination and disease events, the model was structured as a Markov model with a cycle length of 1 week (the average period between an infection and its resolution [30]) over a 5-year time horizon. Figure 1 presents the schematic and natural history model of rotavirus infection. We assumed that the immunity conferred by natural infection or vaccines is imperfect (or partial) so that resolved infections or vaccination lead to 'partially immune' status (i.e. immunity does not fully protect against rotavirus infection but reduces the severity of rotavirus infection outcomes to some extent). We also assumed that infants are exposed to rotavirus infection starting from birth but that infections during the first 2 months (i.e., before the first vaccine dose) are mild or asymptomatic (probably due to maternal antibodies [30]), not incurring any treatment costs. Thus, for the first two months of life before the first vaccine dose, for both vaccinated and unvaccinated strategies, model outcomes were dichotomized using a decision tree–susceptible versus 'infected (partially immune)'–according to whether infants got infected with rotavirus. At the age of 2 months, infants were given the first vaccine dose or not according to each strategy and entered the Markov model, which includes several health-related states: susceptible; infected with wild type rotavirus (asymptomatic, mild, and severe); partially immune after (the first, second, third, or fourth) natural infections; partially immune by vaccination; rotavirus diarrhea-specific death; and death due to other causes (Figure 1). Of the vaccinated infants, uninfected infants entered into the "partially immune by vaccination" state while previously infected infants entered into the "partially immune by first infection." Under the no vaccination strategy, susceptible infants entered the "Susceptible" state and previously infected infants the "partially immune by first infection" state. Individuals then moved between health states according to the natural history model of rotavirus infection with different probabilities depending on their vaccination status, age at infection, number of previous infections, and vaccine efficacy (Table 1). Specifically, the transition probabilities were estimated based on the following base-case assumptions: (1) vaccinated infants received the second dose at the age of 4 months but the period between the first and second doses provides the same level of immunity as a full-dose course (although some clinical trials [31] report different incidence rates between the inter-dose period and the period after the 2nd dose, we assumed the same level of vaccine efficacy for the two different periods since the evidence from the literature is difficult to interpret); (2) vaccinated children may face risks of rotavirus infection due to the imperfect nature of vaccine immunity, but given the same number of previous infections, their risk of infection and proportion experiencing severe rotavirus gastroenteritis are lower than in unvaccinated children [31,32]; (3) for both vaccinated and unvaccinated children, subsequent infections have lower incidence rates and less severe outcomes compared with preceding infections (e.g., the proportion of severe cases caused by a second infection is lower than that by a primary infection and subsequent infections after the second one do not lead to severe cases [8]) (Table 1); and (4) immunity from either natural infection or vaccination lasts over the full time horizon of 5 years, but the level of immunity depends on the number of previous infections. We explored the impact of waning vaccine-induced immunity in sensitivity analyses.

**Table 1:** Model input parameter values and ranges

Parameters	Baseline estimates	Ranges^a^	Distributions^b^	Sources
**Epidemiological parameters**				
5-year cumulative numbers of rotavirus gastroenteritis death (for a 2004 birth cohort)	1,673	1,514–1,833^c^	Not varied	[38]
Estimated age-specific incidence rate of rotavirus diarrhea associated deaths (per 100,000 per month)^d^			Not varied	[37] Estimated
age 0–1 month	0.0	Not varied		
age 2 months	3.5	2.8–4.2		
age 3–5 months	3.8	3.0–4.5		
age 6–8 months	6.5	5.2–7.8		
age 9–11 months	12.2	9.7–14.6		
age 12–14 months	15.3	12.2–18.3		
age 15–17 months	14.0	11.2–16.8		
age 18–23 months	7.9	6.3–9.5		
age 24–35 months	3.4	2.7–4.1		
age 36–47 months	2.9	2.3–3.5		
age 48–59 months	2.6	2.1–3.1		
Cumulative probability of primary infection (by 2 months of age), %	3	0–8	Not varied	Estimated
Ratio of numbers of hospitalizations to deaths attributable to rotavirus gastroenteritis	20.7	10.35–31.05	Not varied	[13]
Ratio of numbers of outpatient visits to deaths attributable to rotavirus gastroenteritis	96.6	32.2–103.0	Not varied	Assumed
Ratios of annual incidence of different rotavirus infection outcomes (without vaccination)				
Primary infections				
- mild vs. severe cases^e^	4.3	2.15–6.45	Not varied	[8]
- asymptomatic vs. severe cases^e^	4.1	2.05–6.15	Not varied	[8]
Secondary infections				
- mild vs. severe cases^e^	21.5	10.8–32.3	Triangular	[8]
- asymptomatic vs. severe cases^e^	19.0	9.5–28.5	Triangular	[8]
Secondary vs. primary severe infections	0.167	0.125–0.209	Not varied	[8]
Tertiary vs. primary 'any' infections^f^	0.478	0.239–0.717	Triangular	[8]
Quaternary vs. primary 'any' infections^f^	0.372	0.186–0.558	Triangular	[8]

**Vaccine characteristics**				
Vaccine coverage (2 doses)	94	90–98	Triangular	[13]
Serotype-specific vaccine efficacy (against severe gastroenteritis), %			Not varied	
G1P[8]	90.8	70.5–98.2^c^		[26]
G3P[8], G4P[8], G9P[8]	86.9	62.8–96.6^c^		[26]
G2P[4] and other combinations of G and P	45.4	0–85.6		[26]
Vaccine efficacy against severe gastroenteritis, % (adjusted for the serotype distribution)	77	43–99	Triangular	Estimated
Vaccine efficacy against mild gastroenteritis, % (not adjusted for the serotype distribution)	41	21–62	Triangular	Estimated

**Costs**^g^				
***Vaccination costs***				
Vaccine price (per dose)	5	0.1–10	Triangular	Assumed
Vaccine wastage rate, %	10	0–20	Triangular	Assumed
Delivery costs^h ^(per dose)	0.7	0.35–1.05	Triangular	Estimated
***Disease treatment costs***				
Outpatient visit (per episode)	3.49	2.88–4.11	Triangular	[13]
Hospitalization (per episode)	19.97	14.98–24.96	Triangular	[13]
***Direct nonmedical costs***				
Transport				
- for outpatient visit	0.69	0.52–0.86	Triangular	[13]
- for hospitalization	4.46	3.35–5.58	Triangular	[13]
Caregiver's time				
- for outpatient visit	2.82	2.12–3.53	Triangular	[13]
- for hospitalization	7.86	5.90–9.83	Triangular	[13]

Discount rate, %	3	0–6	Not applicable	Assumed
Disability weight for diarrhoeal episode	0.119	0.086–0.152	Triangular	[47]

a This column shows the ranges of parameters that were varied during one-way sensitivity analyses.

b This column shows parameters that were varied during the probabilistic sensitivity analysis and distributions assigned to them. Since data often did not contain enough information from which to estimate distribution parameters, triangular distributions were chosen for all parameters.

c 95% confidence interval.

d Monthly incidence rates were converted to weekly transition probabilities within the model, assuming an exponential relationship between the cumulative incidence at different time (age) intervals and time (age). For details, see Additional file 1, which provides a more detailed explanation of the steps taken.

e 'Severe cases' means the aggregate number of outpatient visits, hospitalization, or disease-specific deaths attributable to rotavirus gastroenteritis.

f 'Any' infection includes severe cases, mild cases, and asymptomatic cases.

g All costs are expressed in 2004 US dollars.

h Delivery cost is defined as any costs incurred in distributing and administering vaccines (other than costs for vaccine purchase), including all capital costs (e.g., cold chains and vehicles) and shared operational costs (e.g., salary for personnel, transportation, maintenance, training, social mobilization, and surveillance).

The model tracked the cumulative numbers of different types of rotavirus infection outcomes over the time horizon of 5 years. The model then translated the disease outcomes into DALYs following the standard Global Burden of Disease (GBD) approach [33]. Health losses associated with nonfatal outcomes (i.e., outpatient clinic visits or hospitalizations) were calculated using the disability weights of 0.119 for an episode of diarrhea in general from the GBD study and assuming a duration of 6 days [19]. For the base-case, we did not weight healthy years lived differentially by age, but we did consider age-weighting in a sensitivity analysis for comparability with previous studies.

### Model input

#### Vaccine efficacy

To estimate the vaccine's efficacy against rotavirus gastroenteritis in Vietnam, we used the serotype-specific efficacy data of Rotarix^® ^from the clinical trials performed in Latin American countries (Table 1) [26], and computed an average efficacy based on Vietnam's distribution of rotavirus genotypes. Genotypes of rotavirus are determined by an independent combination of two different groups of proteins, G and P, with the 5 most prevalent genotypes worldwide being G1P[8], G1P[8], G2P[4], G3P[8], G4P[8], and G9P[8] [34]. According to recently published sentinel hospital surveillance data for 1998–2003 [35], the genotype distribution of rotavirus in Vietnam is as follows: G1P[8] (44.5%), strains sharing only P[8] with the vaccine (i.e., G3P[8], G4P[8], or G9P[8]) (26.3%), strains sharing only G1 (2.6%), and strains sharing neither antigen (e.g., G2P[4]) (26.6%). The serotype-adjusted efficacy against severe rotavirus infection was 77%. We assumed an average vaccine efficacy of 41% against mild rotavirus infections based on published literature [8]. In a sensitivity analysis on waning vaccine-induced immunity, we assumed that vaccine efficacy against severe cases would decline during each of the first 5 years of life. In the absence of direct evidence on waning immunity, we applied a conservative estimate of declining vaccine efficacy over age by extending the 2-year efficacy data from Linhares et al.'s study [36] among Latin American infants. The resulting vaccine efficacy estimates (adjusted for serotype distribution) for the first 5 years of life were as follows: 77%, 66%, 57%, 49%, and 43% for age 0, 1, 2, 3, and 4, respectively.

#### Vaccine coverage

Since rotavirus vaccines can be administered with other traditional childhood vaccines [10], we assumed that rotavirus vaccines are administered in Vietnam together with the first and second doses of diphtheria-tetanus-pertussis (DTP) vaccine. The coverage for the first two doses of the DTP vaccine in Vietnam was about 94% and 93%, respectively, in 2004 [13]. For simplicity, we assumed that infants who received the first dose would also complete the second dose, using 94% coverage for the base-case.

#### Incidence of rotavirus infection

We assumed that most children in Vietnam experience rotavirus infection through the fecal-oral route by the age of 5 [37]. We obtained data on cumulative age distribution of hospitalized children due to rotavirus gastroenteritis in Vietnam (under no vaccination) from a published study that reports sentinel surveillance data [37] (Table 1). Because similar data are not available for outpatient visits or deaths due to the virus, for the two forms of severe cases, we assumed the same cumulative age distribution as the one for hospitalization. For unvaccinated children, age-specific, rotavirus-associated mortality rates were estimated by applying the cumulative age distribution to the World Health Organization (WHO) estimate of the total number of rotavirus-associated deaths among Vietnamese children aged < 5 years in 2004 [38] (Table 1). Age-specific incidence rates of rotavirus-associated hospitalization were calculated by applying the estimated rotavirus-associated mortality to the ratio of the projected cases of rotavirus-associated hospitalization and rotavirus diarrhea-specific deaths (~21:1), which was obtained from a previous study in Vietnam [13] (Table 1). The incidence of outpatient visits was estimated using the same approach, but the corresponding ratio was adjusted in the model to improve internal consistency (i.e., the value of the ratio parameter was varied over a plausible range and adjusted so that the model projected the cumulative probability of rotavirus infection, and the age distribution of the severe cases were consistent with the model input and assumptions). Similarly, the incidence rates of mild and asymptomatic infections were derived by applying the above estimated incidence of severe cases (i.e., aggregate incidence of rotavirus-specific deaths, hospitalizations, and outpatient visits) to the ratios of each infection outcome's incidence to the severe outcome's incidence, which were estimated from findings of a cohort study that followed Mexican children up to 2 years of age and provided incidence rates of various rotavirus infection outcomes (overall, severe, mild, and asymptomatic infections) differentiated by the number of previous infections [8] (Table 1). Based on the findings of the same cohort study [8], we assumed that approximately 86% of the aggregate number of severe cases would occur among primary infections and the rest among subsequent infections, and the related incidence rates were adjusted correspondingly. Varying degrees of partial immunity by natural infection were reflected in the incidence of various infection outcomes differentiated by the number of previous infection [8]. The corresponding incidence rates among vaccinated children were back-calculated using the formula for calculating vaccine efficacy, (1-relative risk) × 100. More details on these calculations are provided in Additional file 1.

#### Costs

Adopting a societal perspective in the base-case analysis, we included direct medical costs, which consist of program costs (vaccine and delivery costs) and medical treatment costs, and direct non-medical costs (travel costs, caregivers' time spent caring for sick children). In our analysis conducted from the health care system perspective we included direct medical costs only. All costs were expressed in 2004 US dollars. To estimate vaccine costs, we used an ingredients approach, multiplying the annual number of units of resources consumed by their unit price [39]. Currently, the price of Rotarix^® ^has not yet been set in any developing countries. However, given the manufacturer's stated commitment to differential pricing for developing countries and the recently agreed tiered price of $7/dose in Brazil, a middle-income country with GDP per capita of about $8,000, there is a possibility that the vaccine price per dose in developing regions might be set lower than $7 [40]. Thus, for the base case, we used the price of $5 per dose (Table 1). We assumed a 10% wastage rate of vaccine but did not consider reserve stock [39]. We estimated delivery costs (defined in our analysis as any costs incurred in distributing and administering vaccines other than the cost of the vaccine itself) using the per-dose delivery cost projected for 2004 in the Vietnamese Expanded Program of Immunization (EPI) program budget [41] and multiplying the estimates by the number of doses for a complete course of Rotarix^® ^(Table 1). We obtained data for direct medical (other than program costs) and non-medical costs from Fischer et al.'s study [13], which provides comprehensive estimates of disease-specific treatment costs and caregivers' time and travel costs for different levels of health facilities in rural versus urban areas in Vietnam [42,43]. We estimated the national-level average for each cost item, by calculating a weighted mean of the estimates reported by Fischer et al., accounting for the rural (75%) and urban (25%) breakdown in Vietnam [13] (Table 1). We did not consider the costs for treating adverse events since the adverse events attributable to rotavirus vaccines are reported to be negligible [26].

### Uncertainty analysis

To explore uncertainty around parameters, we conducted univariate sensitivity analysis over the plausible ranges of key parameters (Table 1). As an extension of the univariate sensitivity analysis we performed a threshold analysis to identify the maximum per-dose vaccine price at which vaccination would still have a cost-effectiveness ratio below the per-capita GDP in Vietnam. We also performed a multivariate, probabilistic sensitivity analysis each perspective, based on 10,000 Monte Carlo simulations (TreeAge 2006) drawing parameter values from the distributions specified in Table 1. The results were used to construct a cost-effectiveness acceptability curve from each perspective.

## Results

### Health outcomes

**Figure 2:**
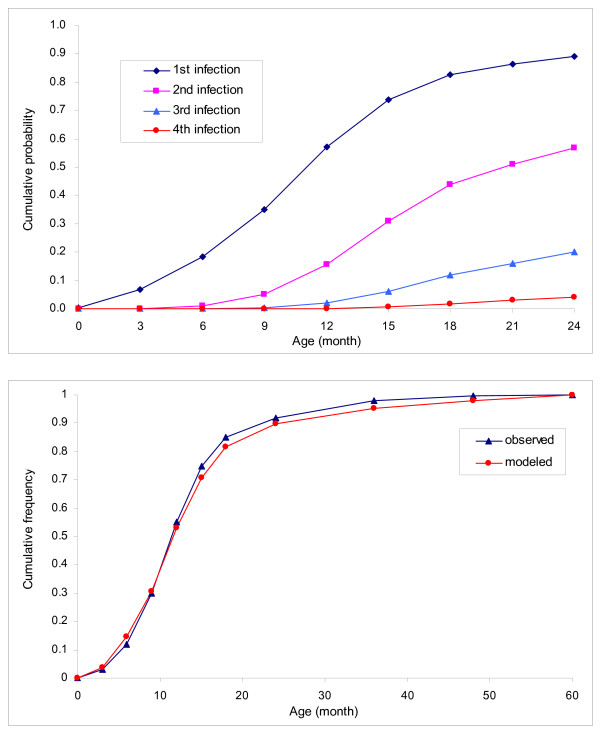
**Selected model-predicted health outcomes**. The upper panel presents the cumulative probabilities of the primary and subsequent rotavirus infections over time under no vaccination. The lower panel shows the cumulative age distribution of hospitalized cases associated with rotavirus gastroenteritis (observed data versus model estimates).

In the absence of vaccination, 58% of infants would get infected during the first year of life. With 94% vaccination coverage, approximately 47% of children would get infected with rotavirus (including asymptomatic infections) before age 5, and the incidence of severe cases of rotavirus gastroenteritis would be reduced by about 67%. Both vaccinated and unvaccinated children would experience about two rotavirus infections on average (~2.0 times for vaccinated and ~2.3 times for unvaccinated children) by 5 years of age. Assuming 94% coverage, vaccinating a cohort of 1,644,000 Vietnamese infants would prevent 105,300 outpatient visits, 22,600 hospitalizations, and 1,090 premature deaths attributable to rotavirus infection over a five year period, compared to no intervention (undiscounted) (Table 2). Figure 2 (upper panel) shows the estimated cumulative probabilities of the first, second, third, and fourth infections, which have a similar pattern to the ones reported in the previously published cohort study [8]. Figure 2 (lower panel) presents the cumulative age-distribution of hospitalized cases, which is closely fitted to the observed data [35]. Translating the health outcomes into summary measures of healthy life, vaccination would avert 31,600 DALYs (discounted at 3% per annum) (Table 2).

**Table 2:** Base-case results

	Societal perspective		Healthcare system perspective	
	
	No vaccination	Routine vaccination	No vaccination	Routine vaccination
Total cases of outpatient visits (thousands)	158.0	52.7	158.0	52.7
Total cases of hospitalization (thousands)	33.9	11.3	33.9	11.3
Total cases of premature deaths	1,640	550	1,640	550
Cost (2004 $) (thousands)	1,973	18,906	1,151	18,577
Incremental cost (2004 $) (thousands)	--	16,933	--	17,462
Effectiveness (DALYs) (thousands)	1,887	1,856	1,887	1,856
Incremental effectiveness (DALYs averted) (thousands)	--	31.6	--	31.6
ICER ($/DALY averted)	--	**540**	--	**550**

DALY: disability-adjusted life year. ICER: incremental cost-effectiveness ratio

### Cost-effectiveness: base-case analysis

Assuming a vaccine price of $5 per dose (with 10% wastage) and 94% coverage, the average vaccination cost per vaccinated child with 2 doses of rotavirus vaccine would be $11.10, and the total program costs for the 2004 birth cohort would be $18.2 million. From the societal perspective, accounting for medical treatment costs and direct non-medical costs in addition to the program costs, the total costs were $19.0 million with vaccination and $2.0 million without vaccination. From the health care system perspective, considering direct medical costs only, the corresponding total lifetime costs were $18.6 million and $1.2 million, respectively. Accordingly, combining both health and cost outcomes, the estimated incremental cost per DALY averted of routine vaccination would be $540 from the societal perspective and $550 from the health care system perspective (Table 2).

### Uncertainty analysis

**Figure 3:**
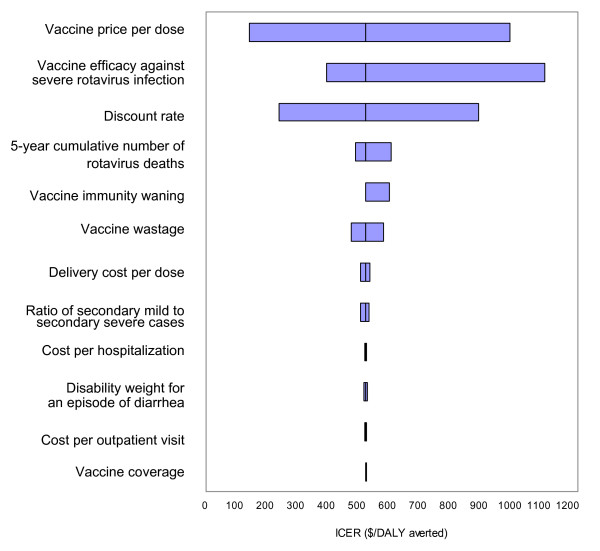
**Selected results of one-way sensitivity analyses**. This graph presents selected results of univariate sensitivity analyses from the societal perspective. The x-axis represents the ranges of the incremental cost-effectiveness ratios of infant rotavirus vaccination in Vietnamese children when the baseline estimates of several key parameters were varied over plausible ranges. The vertical line represents the base case incremental cost-effectiveness ratio of rotavirus vaccination.

In univariate analyses, results were most sensitive to vaccine price, vaccine efficacy against severe gastroenteritis, and discount rate (Figure 3). For example, when the vaccine unit price was set at $1, the incremental cost-effectiveness was $100/DALY averted, but the corresponding value was $1,080/DALY averted with a vaccine price of $10 per dose. The corresponding ratio was $755/DALY averted when the price was set at $7 per dose (which is the recently agreed tiered price of Rotarix^® ^in Brazil). In the threshold analysis for the vaccine price, the program would be cost-effective based on a benchmark of $580/DALY averted (equal to the per-capita GPD of Vietnam) at prices up to $5.41 per dose, and would be cost-effective based on a benchmark of $1,740/DALY averted (three times GDP per-capita) at prices up to $32.4 per dose. When vaccine efficacy against severe rotavirus gastroenteritis was varied between 43% and 99%, the incremental cost-effectiveness ratio ranged from $1,200 to $410 per DALY averted. With no discounting, the incremental cost-effectiveness ratio was $220/DALY averted; with a discount rate of 6% the ratio increased to $960/DALY averted. Results were moderately sensitive to rotavirus-associated mortality, waning vaccine-induced immunity, and vaccine wastage rate. If vaccine-induced immunity waned over five years, corresponding to a decline in age-specific vaccine efficacy from 77% in year 1 to 43% in year 5, the incremental cost per DALY changed by approximately 15%, increasing from $540 (base-case assuming no waning) to $630. The general results were also robust to delivery cost, cost per hospitalization or outpatient visit, and the disability weight for diarrhea (Figure 3).

**Figure 4:**
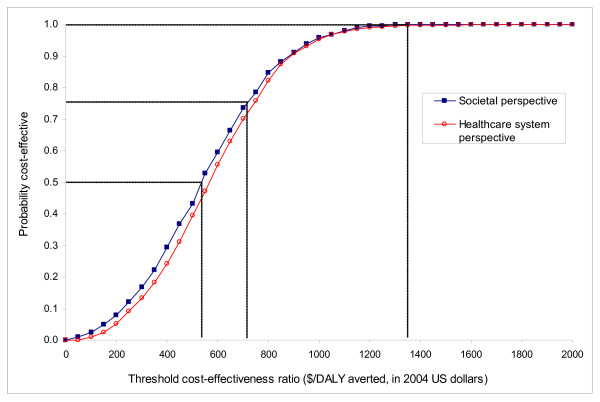
**Cost-effectiveness acceptability curves**. This figure summarizes the results of probabilistic sensitivity analysis from the societal perspective. The curve shows the probabilities that rotavirus vaccination is cost-effective at varying cost-effectiveness threshold ratios.

Figure 4 presents the results of multivariate uncertainty analysis in the form of a cost-effectiveness acceptability curve from each perspective. The curve from the societal perspective shows that the probabilities that the program would be cost-effective are 50%, 75%, and 100% at thresholds of 530, $710, and $1,350 per DALY averted, respectively.

## Discussion

The epidemiology of rotavirus infection has been considered relatively simple compared to that of other pathogens with multiple transmission routes (e.g., HIV, human papilomavirus, or hepatitis B virus), although many aspects of rotavirus natural history are unknown and uncertain. Thus, most previous studies have evaluated the impact of rotavirus vaccines using a simple static model, not fully incorporating all key features of rotavirus infection, and the potential impact of the features that are not incorporated in the models has remained unexplored. Being motivated in part to examine such impact on the cost-effectiveness of rotavirus vaccines in low-income countries, we developed a Markov model that allows more flexibility in reflecting the key features of rotavirus infection and exploring the uncertainty surrounding them. With a time interval that approximately corresponds to an episode of rotavirus infection, our model explicitly considers the timing of events and, unlike previous studies performed in low income country settings, allows for reinfections (up to four), taking into account partial immunity by wildtype infection that varies depending on the number of previous infections.

As such, our model enables more detailed simulations of the natural history and epidemiology of rotavirus. Our model tracks the number of children who experience different numbers of rotavirus infections over time, and thus provides cumulative probabilities (e.g., Figure 2, upper panel) of various types of infection outcomes and cumulative age distribution (e.g., Figure 2, lower panel) as well as overall cases of health outcome measures (Table 2). Our results show that children may get infected with wildtype rotarivus multiple times over the 5-year horizon, but that a majority (~84% under the base-case assumptions) of severe gastroenteritis would occur during the first infection. Our results also show that about 20% of infants would experience rotavirus infection by 6 months of age (Figure 2, lower panel). These findings reaffirm the importance of a strict adherence to the recommended immunization schedule (2 and 4 months of age) once rotavirus vaccines are introduced. Further, it is noteworthy that the model-predicted percentage of reduction in severe cases, 67%, is lower than the initially assumed average vaccine efficacy, 77%, but is a more realistic assessment of the avertable disease burden since it was predicted using the model incorporating more realistic coverage, competing mortality, and age-dependency of disease severity.

When we added vaccine effects to the natural history model, our results suggest that, although the rotavirus vaccine would not completely protect against rotavirus infection itself due to the imperfect nature of vaccine immunity and common rotavirus infection in Vietnam, infant vaccination would significantly reduce the disease burden of severe gastroenteritis caused by the virus. Under the base-case assumptions, the incremental cost per DALY averted was $540 from the societal perspective. The value is higher than those of many other traditional childhood vaccines administered in developing regions (e.g.,~$30 [in 2000 international dollars] per DALY averted for measles vaccination with 80% coverage in sub Saharan region [44]), but is still close to Vietnam's 2006 GDP per capita of about $580 (2004 US dollars) [45], which is used as a cost-effectiveness threshold in our analysis [29]. Accordingly, our findings show that a rotavirus vaccination program may be considered *very cost-effective *in Vietnam based on the commonly used GDP per capita benchmark [29].

When compared to a previous study performed by Fischer and colleagues in the same setting [13], our model's point estimate of the cost per DALY averted is approximately 6-fold higher than the corresponding value estimated by Fischer et al., of $87/DALY averted (in 2004 US dollars, assuming a societal perspective and a vaccine price of $5 per dose; calculated based on the incremental costs and effectiveness from the societal perspective reported in Table 3 in Fischer et al.'s study [13]). Given that we obtained most cost data from Fischer et al.'s study making very similar assumptions on resource use, some possible sources of the discrepancy include that: (1) for 5-year cumulative number of rotavirus associated deaths, we used a more recent WHO estimate which was much lower than the one used in Fischer et al.'s study (1,673 versus 6,050 cases); (2) we assumed a 3-fold higher value (i.e. 96 versus 32) for the ratio of outpatient visits to rotavirus deaths; (3) we used a lower efficacy (i.e. 77% versus 93% against hospitalization and 78% against outpatient visits) adjusted for the local genotype distribution; (4) we used a Markov model, unlike Fischer et al.'s study, distinguishing between primary and subsequent rotavirus infections and considering immunity due to wildtype infections. Our sensitivity analysis results suggest that a significant portion of the differences may be due to the differences in the key epidemiological parameters. However, further research to quantify the contribution of each factor to the discrepancy may provide more insight into the impact of different sources of uncertainty on the cost-effectiveness rotavirus vaccines.

Our study has several limitations. First, although our model attempted to reflect the natural history of rotavirus infection more comprehensively, we did not model seasonality or coinfection by different serotypes of rotavirus. Second, local epidemiological data quality was variable, and country-specific data were not always available. We had to estimate values of some key epidemiological parameters (e.g., genotype-specific vaccine efficacy and incidence rates of primary and subsequent rotavirus infections) based on data from other countries. Third, our model is static and does not capture the effects of vaccination on the force of infection (the rate at which susceptible individuals get infected) in populations over time. Fourth, we did not include Rotateq^®^, another new rotavirus vaccine, in our analysis. Given that Rotateq^® ^can cover more serotypes circulating in Vietnam, this vaccine may provide a higher serotype-adjusted vaccine efficacy against severe rotavirus diseases. However, a majority of the effectiveness gains from the higher vaccine efficacy may be offset by higher vaccine costs. Accordingly, we expect similar results with Rotateq^®^. Fifth, while we conducted a sensitivity analysis to explore the impact of waning vaccine-induced immunity and found an approximate 15% increase in the cost-effectiveness ratio, we did not explore the implications of specific relationships and correlations between natural immunity and vaccine induced immunity within individuals. As better data become available, these will be important to incorporate to ensure the impact of vaccination is not overestimated. Finally, it was not possible to project the potential impact of routine vaccination on the serotype distribution of rotavirus. Because the currently available rotavirus vaccines do not provide cross-immunity for all serotypes, and because serotypes of rotavirus are known to vary over time even in the same country [3], if routine vaccination affects the dynamics of serotype evolution in some way, future vaccine efficacy may in turn be affected considerably.

Despite the limitations, our study provides insight into future research areas for rotavirus disease control using vaccines. Our analysis highlights that there is a high level of uncertainty and variability around rotavirus epidemiology and vaccine efficacy. For example, in adjusting vaccine efficacy for the genotype distribution of rotavirus, the genotype distributions differed widely across studies even within Vietnam [35,37,46], leading to a wide range of adjusted vaccine efficacy between 46% [37] and 81% [46] with the base-case rate of 77% [35]. High uncertainty also exists around the level of immunity by natural infection in Vietnam, given that no other cohort study comparable to Velazquez et al.'s study in Mexico [8] (from which we obtained the estimates of relative risks among various rotavirus infection outcomes) has been conducted in different settings with different genotype distributions. In addition, little is known as to how long immunity conferred by vaccines or wild type infections would last. All these warrant further research on the genotype-specific vaccine efficacy and possible waning of vaccine immunity in local settings and more comprehensive surveillances to monitor potential genotype evolution following vaccine introduction. Particularly for Vietnam, given that the base-case incremental cost-effectiveness ratio ($540/DALY averted) was very close to Vietnam's per capita GDP (~$580), which is often used as a surrogate threshold for identifying *very cost-effective *interventions, and that rotavirus vaccines are likely to compete against other childhood vaccines for limited budget allocations, it may be worth performing a value of information analysis in order to set priorities among such future studies. Finally, as our comparison exercise with a previous study implies, it may be worthwhile to examine uncertainty related to different model type or structure more formally.

## Conclusion

In conclusion, introducing rotavirus vaccines would be a cost-effective public health intervention in Vietnam and other developing countries with high rotavirus disease burden. However, given the high uncertainty about vaccine efficacy and potential changes in rotavirus epidemiology that might in turn affect rotavirus vaccine efficacy, further clinical research is warranted and a re-evaluation of a rotavirus vaccination program from a long-term perspective may be necessary as new information emerges.

## Competing interests

The authors declare that they have no competing interests.

## Authors' contributions

SK, SG, and JS conceptualized and designed the study. SK acquired data and performed analyses. SK, SG, and JS interpreted the data and results. SK drafted the manuscript. SG provided administrative and technical support, and JS provided statistical expertise. All authors contributed to the revision of the manuscript and approved the final version.

## Pre-publication history

The pre-publication history for this paper can be accessed here:



## Supplementary Material

Additional file 1 **Estimation of incidences of rotavirus infection outcomes.** The document describes the steps taken to estimate incidence rates of rotavirus infection outcomes and to convert the incidence rates to transition probabilities. Click here for file
