# Supplementary material for: Cost-effectiveness of Rotavirus vaccination in Vietnam
Source: BMC Public Health. 2009 Jan 21;9:29. doi: 10.1186/1471-2458-9-29 (PMC2663769; doi:10.1186/1471-2458-9-29)
Supplement: Additional file 1 — Estimation of incidences of rotavirus infection outcomes. The document describes the steps taken to estimate incidence rates of rotavirus infection outcomes and to convert the incidence rates to transition probabilities. [file 1471-2458-9-29-S1.doc]

**Cost-effectiveness of rotavirus vaccination in Vietnam**

Sun-Young Kim, Sue J. Goldie, Joshua A. Salomon

**Estimation of incidence of rotavirus infection outcomes**

**Clinical definitions and assumptions**

- We followed the clinical definition of severe gastroenteritis (an episode of diarrhea requiring overnight hospitalization or rehydration therapy in a medical facility) used in a phase III clinical trial of Rotarix® [1].
- Based on the above definition, we assumed that rotavirus infection leads to one of the following outcomes: asymptomatic infection, mild gastroenteritis (requiring home care only), or severe gastroenteritis (requiring medical treatment).
- Severe gastroenteritis was subdivided into three categories: outpatient visit, hospitalization, or death.
- We assumed that reinfections of rotavirus occur, but that subsequent infections after the second one do not lead to severe cases.

**Key data sources and related assumptions**

- The number of rotavirus-associated deaths in children ages 0-4 years in Vietnam in 2004, 1,673, was obtained from a World Health Organization document [2].
- The data on cumulative age distribution of hospitalized children due to rotavirus gastroenteritis in Vietnam (under no vaccination) was obtained from a published study reporting sentinel surveillance data from several hospitals [3]. Since analogous data were not available for the other two types of severe cases—rotavirus associated deaths and outpatient visits, we assumed that ***rotavirus associated deaths and outpatient visits have the same cumulative age distribution as that for hospitalization***. The numerical values of the age-specific cumulative probability of rotavirus associated deaths (or severe rotavirus cases) that were used in the model are shown in column (c) in Table A1.
- Based on the findings of the cohort study by Velazquez et al. [4], which followed Mexican children up to 2 years of age and provided incidence rates of various rotavirus infection outcomes (overall, severe, mild, and asymptomatic infections) differentiated by the number of previous infections, we assumed that approximately 86% of the aggregate number of severe cases would occur among primary infections and the rest among subsequent infections.

**Calculation of age-specific incidence of rotavirus associated deaths during the primary infection (among unvaccinated children)**

- Step 1: The age distribution of rotavirus associated deaths was calculated by multiplying the difference in cumulative probability of rotavirus deaths between adjacent ages (column (d) in Table A1) by the total number of rotavirus deaths among children aged 0-4 years, 1,673. The resulting figures are shown in column (e).
- Step 2: In order to differentiate the number of severe cases between the primary and secondary infections, the figures in column (e) were multiplied by 0.86, which is the estimated fraction of all severe cases that follow from the first infection. See column (f) in Table A1 for the calculations.
- Step 3: To estimate the age-specific numbers of hospitalizations and outpatient visits attributable to rotavirus infection, we obtained initial estimates of the following parameters from Fischer et al.’s study [5]:
  - Ratio of hospitalized cases to deaths (parameter name: ratio_hosp_death): 20.74
  - Ratio of outpatient visits to deaths (ratio_out_death): 32.19 (🡪 *Note: as described in Step 12 below, this initial value was later adjusted to fit empirical data on the cumulative probability of infection at ages 24 months and 60 months, and the cumulative probability of severe infection by age*)

For each age interval, the initial numbers of hospitalizations and outpatient visit were calculated by multiplying the number of rotavirus associated deaths, the figures from column (f), by 20.74 and 32.19, respectively. 🡪 Results were recorded in columns (g) and (h).

- Step 4: The estimated number of severe cases was calculated as the row sum of the figures from columns (f), (g), and (h). 🡪 Results were recorded in column (i).
- Step 5: To calculate the numbers of mild and asymptomatic cases, we also obtained from Valezquez et al. [8] estimates of the following parameters:
  - Ratio of mild to severe cases during the primary infection (ratio_mild1_severe1): 4.333
  - Ratio of asymptomatic to severe cases during the primary infection (ratio_asympt1_severe1): 4.083

For each age interval, the initial numbers of cases of mild and asymptomatic infection were calculated by multiplying the numbers of severe cases, the figures from column (i), with 4.333 and 4.083, respectively.

- Step 6: The estimated cases of *any* rotavirus infection was calculated as the row sum of the figures from columns (i), (j), and (k). 🡪 Results were recorded in column (l).
- Step 7: For each age interval, the incidence rate of rotavirus associated death was calculated by applying the number of rotavirus associated deaths (from column (f)) to the size of susceptible (uninfected) population. The size of the susceptible population for age group *t* was estimated by subtracting the number of all cause deaths (obtained from a life table for Vietnamese children [6]) and the number of *any* infections for age group *t-1* from the susceptible population size for age group *t-1*. 🡪 Results were recorded in column (m).
- Step 8: The incidence rates of mild and asymptomatic rotavirus infections were calculated in a similar way. The incidence of *any* rotavirus infection was assumed to be the sum of the incidence rates for severe, mild, and asymptomatic cases for a given age group.
- Step 9: To estimate the incidence rate of severe cases during secondary infections, we first obtained the approximate value of the following parameter from Velazquez et al. [4]:
  - Ratio of severe cases occuring after secondary infection to severe cases occuring after the primary infection (ratio_severe2_severe1): 0.167 (🡪 Note that this value is calculated using the fraction of severe cases among all severe cases, 0.86. Specifically, the ratio is (1-0.86)/0.86)

The incidence rate of severe cases for the secondary infection was then calculated by multiplying the corresponding incidence rate for the primary infection, column (m), by the ratio describe above, 0.167.

- Step 10: The incidence rates of mild and asymptomatic cases were calculated by multiplying the incidence from Step 9 by the estimates of the following parameters, which were obtained from Velazquez et al. [4]:
  - Ratio of incidence of secondary mild to severe cases (ratio_mild2_severe2): 21.5
  - Ratio of incidence of secondary asymptomatic to severe cases (ratio_mild2_severe2): 19.0
- Step 11: The incidence rates of any type of tertiary (3^rd^) and quaternary (4^th^) rotavirus infection were calculated by multiplying the incidence for the primary infection (from Step 8) by the estimates of the following parameters, which were also obtained from Velazquez et al. [4]:
  - Ratio of incidence of any 3rd infection to any 1st infection (ratio_any3_any1): 0.478
  - Ratio of incidence of any 4th infection to any 1st infection (ratio_any4_any1): 0.372
- Step 12: We fit our incidence model described above to the two empirically reported data points of cumulative probability of rotavirus infection—about 90% by the age of 24 months and about 100% by the age of 60 months. In fitting the incidence model, because of the lack of further empirical data that were not already used in estimating model input, we were not able to take a formal approach to multi-parameter model calibration. Instead, according to our judgment that the ratio of cases of outpatient visits to deaths (ratio_out_death) is one of the most uncertain parameters based on available empirical studies, we chose to vary this parameter until the model yielded an approximate fit to the empirical data points (for external validity), while also adhering to epidemiological constraints on model inputs. As the parameter value was varied, the calculations described in Step 3 to Step 11 were repeated.
- Step 13: After iterating this process, the base-case value of the parameter, *ratio_out_death*, was determined to be 96.6 (approximately three times the initial value, 32.19). The final base-case values of relevant model input based on this parameter value, 96.6, are shown in columns (g) through (m).

**Calculation of age-specific incidence of rotavirus associated deaths during the primary infection (among vaccinated children)**

- The corresponding incidence rates among vaccinated children were back-calculated using the formula for calculating vaccine efficacy, (1-relative risk)×100.
- For example, the incidence rate of rotavirus associated death among vaccinated children was calculated as follows:
  - Incidence rate of rotavirus associated deaths among unvaccinated children: rate_death1_NoVax [derived as described above, and reported in column (m)].
  - Base-case vaccine efficacy against severe cases (adjusted for the serotype distribution of rotavirus in Vietnam): Efficacy_severe = 77%
  - rate_death1_Vax = rate_death1_NoVax*(1-Efficacy_severe)

= rate_death1_NoVax*(1-0.77)

**Conversion of weekly incidence rates to weekly transition probabilities**

- Finally, the incidence rates of rotavirus infection outcomes were converted to weekly transition probabilities, assuming an exponential form for cumulative incidence over time.
- For example, the weekly transition probabilities of *any* primary infection among unvaccinated children who survived deaths from other causes is expressed as below:

Prob (transition from susceptible to any primary infection)

= 1-exp(-rate_any1_NoVax)

= 1-exp[-{rate_death1_NoVax + rate_hosp1_NoVax + rate_out1_NoVax + rate_mild1_NoVax + rate_asympt1_NoVax}]

Further, transition probabilities of rotavirus associated deaths are calculated taking into account other competing risks, as follows:

Prob (transition from susceptible to primary rotavirus associated death)

= [1-exp(-rate_any1_NoVax)]*fraction of primary deaths among any primary infections

where, fraction of primary deaths among any primary infections

= rate_death1_NoVax/(rate_death1_NoVax + rate_hosp1_NoVax + rate_out1_NoVax + rate_mild1_NoVax + rate_asympt1_NoVax)

**References**

1. Ruiz-Palacios GM, Perez-Schael I, Velazquez FR, Abate H, Breuer T, Clemens SC et al.: Human Rotavirus Vaccine Study Group. Safety and efficacy of an attenuated vaccine against severe rotavirus gastroenteritis. *N Engl J Med* 2006;354(1):11-22.

2. WHO: Estimated rotavirus deaths for children under 5 years of age: 2004 (<http://www.who.int/immunization_monitoring/burden/rotavirus_estimates/en/index.html>) Accessed on Nov. 8, 2007.

3. Van Man N, Van Trang N, Lien HP et al.: The epidemiology and disease burden of rotavirus in Vietnam: sentinel surveillance at 6 hospitals. *J Infect Dis* 2001;183:1707-12.

4. Velazquez FR, Matson DO, Calva JJ, et al.: Rotavirus infections in infants as protection against subsequent infections. *N Engl J Med* 1996;335:1022-8.

5. Fischer TK, Anh DD, Antil L, Cat ND, Kilgore PE, Thiem VD, Rheingans R, Tho le H, Glass RI, Bresee JS: Health care costs of diarrheal disease and estimates of the cost-effectiveness of rotavirus vaccination in Vietnam. *J Infect Dis* 2005;192(10):1720-6.

# 6. WHO: Life Tables for WHO Member States ([**http://www.who.int/whosis/database/life_tables/life_tables.cfm**](http://www.who.int/whosis/database/life_tables/life_tables.cfm)) Accessed on Nov. 19, 2007.

Table A1. Estimation of incidence of rotavirus associated deaths (for the primary infection) in the absence of vaccination

| Age  in month  (a) | Age  in week  (b) | Cumulative  frequency  (rotavirus  associated  deaths)  (c) | Delta  cumulative  frequency  (d) | Aggregate  number of  rotavirus  associated  deaths by  age group  (e) | Estimated number of  rotavirus associated deaths during  the primary  infection  (f) | Estimated number of  hospitalized  cases during  the primary  infection  (g) | Estimated number of  outpatient  visit during  the primary  infection  (h) | Estimated number of  severe cases  during  the primary  infection  (i)  =(f)+(g)+(h) | Estimated number of  mild cases  during  the primary  infection  (j) | Estimated number of  asymptomatic cases during  the primary  infection  (k) | Estimated number of  cases of  any infection  during  the primary  infection  (l)  =(i)+(j)+(k) | Weekly Incidence rate of  rotavirus associated deaths during the  primary infection  (per 100,000)  (m) |
| --- | --- | --- | --- | --- | --- | --- | --- | --- | --- | --- | --- | --- |
| 0 | 0 | 0.000 | 0.000 | 0 | 0 | 0 | 0 | 0 | 17,358^#^ | 16,357^#^ | 37,721^#^ | 0.00 |
| 2 | 13 | 0.000* | 0.035 | 59 | 51 | 1,053 | 4,905 | 6,009 | 26,037 | 24,535 | 56,581 | 0.82 |
| 3 | 26 | 0.035 | 0.125 | 209 | 179 | 3,721 | 17,326 | 21,226 | 91,979 | 86,673 | 199,878 | 0.88 |
| 6 | 39 | 0.160 | 0.185 | 309 | 266 | 5,519 | 25,697 | 31,482 | 136,420 | 128,550 | 296,451 | 1.51 |
| 9 | 51 | 0.345 | 0.272 | 454 | 391 | 8,105 | 37,742 | 46,238 | 200,366 | 188,807 | 435,411 | 2.84 |
| 12 | 64 | 0.617 | 0.201 | 337 | 290 | 6,017 | 28,017 | 34,324 | 148,737 | 140,156 | 323,217 | 3.56 |
| 15 | 77 | 0.818 | 0.091 | 151 | 130 | 2,700 | 12,572 | 15,402 | 66,742 | 62,891 | 145,035 | 3.27 |
| 18 | 103 | 0.909 | 0.054 | 91 | 78 | 1,627 | 7,574 | 9,280 | 40,212 | 37,892 | 87,383 | 1.85 |
| 24 | 154 | 0.963 | 0.022 | 36 | 31 | 649 | 3,023 | 3,703 | 16,048 | 15,122 | 34,874 | 0.79 |
| 36 | 206 | 0.985 | 0.010 | 17 | 14 | 298 | 1,389 | 1,702 | 7,376 | 6,951 | 16,029 | 0.68 |
| 48 | 253 | 0.995 | 0.005 | 8 | 7 | 149 | 695 | 851 | 3,688 | 3,475 | 8,014 | 0.61 |
| 59 |  |  |  |  |  |  |  |  |  |  |  |  |
| (Sum) |  | 1.000 |  | 1,673 | 1,439 | 29,838 | 138,940 | 170,217 | 737,606 | 695,052 | 1,602,874 |  |

* The cumulative probability of severe rotavirus gastroenteritis for the age interval 0-2 month was forced to be 0 based on the base-case model assumption that rotavirus infection during the first 3 months of life rarely leads to severe cases.

# It was assumed that about 1/3 of the non-severe cases that occurred during the first 6 months of the life (age interval 0-5 months) occurred during the first 3 months (age interval 0-2 months).
